# Effects of Traditional Chinese Medicine Anticancer Decoction Combined with Basic Chemotherapy and Nursing Intervention on Oral Cancer Patients after Surgery and Its Effect on Tumor Markers and Immune Function

**DOI:** 10.1155/2022/6341381

**Published:** 2022-03-30

**Authors:** Dan Jiang, Fengying Xiao, Lihua Liu, Zhen Meng, Chengwei Zhang

**Affiliations:** ^1^Department of Stomatology, Yantaishan Hospital, Yantai 264000, China; ^2^Operation Room, Jiyang People's Hospital, Jinan 251400, China; ^3^Department of ICU, The Affiliated Qingdao Central Hospital of Qingdao University, The Second Affiliated Hospital of Medical College of Qingdao University, Qingdao 266042, China; ^4^Department of Ultrasound, Zhangqiu District People's Hospital, Jinan 250200, China; ^5^Medical Laboratory and Diagnostic Center, Jinan Central Hospital, Jinan 250013, China

## Abstract

**Objective:**

To prospectively study the application effect of traditional Chinese medicine (TCM) anticancer decoction with basic chemotherapy and nursing intervention on oral cancer patients after surgery and the effect on tumor markers and immune function.

**Methods:**

Eighty-four postoperative oral cancer patients in our hospital from May 2017 to February 2019 were selected and divided into observation group (42 cases) and control group (42 cases). The control group was treated with basic chemotherapy combined with basic nursing care, and the observation group was treated with TCM anticancer decoction and comprehensive nursing intervention on the basis of the control group. The clinical efficacy, the occurrence of adverse reactions, the satisfaction of nursing care, and the two-year cumulative survival rate of the two groups were compared. The immune function, tumor marker level, VAS score, QoR40 score, and survival quality score of the two groups were compared before and after nursing care.

**Results:**

The total clinical treatment efficiency of the observation group (88.10%) was significantly higher than that of the control group (69.05%), and the differences between the two groups in oral cleanliness, aspiration frequency, and oral comfort were statistically significant (*P* < 0.05). The differences in the occurrence of halitosis, oral fungal infection, leukopenia, gastrointestinal reaction, and fever in the observation group were statistically significant compared with the control group (*P* < 0.05). The nursing satisfaction rate in the observation group (95.24%) was significantly higher than that in the control group (78.57%). The two-year cumulative survival rate of the observation group (92.86%) was significantly higher than that of the control group (73.81%). After nursing care, CD4+, CD4+/CD8+, VAS scores, QoR40 scores, and quality of survival scores in both groups all increased, and CD8+, CD56+, CEA level, NSE level, and CA19-9 level all decreased (all *P* < 0.05).

**Conclusion:**

The clinical efficacy of TCM anticancer decoction with basic chemotherapy and nursing interventions in the treatment of postoperative oral cancer patients was remarkable, which could significantly improve patients' oral cleanliness and comfort, reduce the frequency of sputum aspiration, improve patients' immunity, reduce tumor marker levels, inhibit tumor activity, improve patients' nursing satisfaction, further improve patients' treatment compliance, reduce patients' pain level, improve patients' survival quality, and prolong patients' survival time with high safety. It could be used as a theoretical basis for subsequent clinical research.

## 1. Introduction

Oral cancer, as a common malignant tumor of the head and neck, accounts for 0.58% to 1.3% of all malignant tumors in the body and is most common in people aged 40 to 70 years, with the incidence increasing year by year and mostly squamous epithelial cell carcinoma [1]. According to relevant data [2–4], about 270,000 people are diagnosed with oral cancer each year, and 2/3 of these patients are found in developing countries. Oral cancer occurs in the oral cavity and any anatomical parts adjacent to it, which can seriously affect patients' normal diet, speech, and other functions, thus affecting their survival quality and even threatening their life safety. Surgical resection is the first choice of treatment for oral cancer, but there are risks such as incomplete surgical resection, easy recurrence after surgery plus possible combination with other serious complications, the prognosis is poor, and the survival rate is extremely low [5].

Some studies have shown that comprehensive treatment such as chemotherapy and Chinese medicine treatment after oral cancer surgery can improve the therapeutic effect of patients after surgery [6, 7]. Although chemotherapy has obvious antitumor effects, long-term treatment can also cause serious adverse effects such as bone marrow suppression and gastrointestinal reactions [8]. Traditional Chinese medicine (TCM) has been wildly used in treatment of various human ailments including cancer [9, 10], neurological disease [11, 12], lung disease [13–15], and infectious disease [16]. Given obvious antitumor effects and good safety, TCM can play a role in increasing the effectiveness and reducing toxicity [17]. However, there are few studies on anticancer soup for the treatment of patients with intermediate and advanced oral cancer after surgery.

Relevant studies have confirmed that pathogenic bacteria can easily colonize and multiply in the oral cavity of the organism, and normal people can effectively remove oral pathogenic bacteria due to their own cleansing function, which makes the incidence of serious infection low [18, 19]. However, postoperative oral cancer patients have surgical trauma in the oral cavity, and the daily secretion of blood and saliva is significantly reduced. Moreover, long-term postoperative fasting makes the self-cleaning function of the patient's oral cavity significantly weakened, and the rapid reproduction of pathogenic bacteria leads to serious infection of the trauma, which in turn affects the effect of surgical treatment. Therefore, a systematic understanding of oral cancer patients' needs, including physical, psychological, and interpersonal communication needs, is very important for postoperative oral cancer patients to adopt effective oral care measures and improve their survival quality. Therefore, this study is aimed at investigating the effects of anticancer soup combined with basic chemotherapy and nursing intervention on tumor markers and immune function of postoperative oral cancer patients.

## 2. Materials and Methods

### 2.1. General Data

Eighty-four cases of postoperative oral cancer patients admitted to our hospital from May 2017 to February 2019 were selected and divided into observation and control groups, 42 cases each.

Inclusion criteria were as follows: (i) patients who met the relevant diagnostic criteria for oral cancer [20]; (ii) patients who were 44-71 years old; (iii) patients who were diagnosed with oral squamous cell carcinoma by postoperative pathological examination and had typical symptoms such as dysphagia, unexplained bleeding, nodules, masses, and white smooth squamous plaques [21]; (iv) patients who underwent tumor resection; (v) patients who had no allergic reactions to the drugs used in this study; (vi) patients with an ASA classification of I to III; and (vii) patients and their families agreed to participate in this study and signed the informed consent form.

Exclusion criteria were as follows: (i) patients who had received antitumor treatment before admission, (ii) patients who combined with primary tumors of nose and throat, (iii) patients with severe organ dysfunction, (iv) patients who were accompanied by serious systemic diseases, (v) patients who combined with malignant tumors of other organs, (vi) patients with poor compliance and active withdrawal from the study, and (vii) treatment plan had to be terminated due to serious adverse reactions during the treatment period. The differences between the two groups were not statistically significant (*P* > 0.05) when comparing baseline data, such as gender, age, and disease type, and were comparable (Table 1). This study was approved by the Medical Ethics Committee of our hospital (LLBH20170316), and all patients gave their informed consent and signed the informed consent form.

### 2.2. Treatment and Nursing Methods

Patients in the control group were treated with basic chemotherapy combined with basic nursing care. The basic chemotherapy regimen was as follows: 1 mg vincristine (National Drug Approval H20068151, Luyi Furen Oncology Drug Co., Ltd., 1 mg) and 30 ml 0.9% sodium chloride solution were intravenously injected in the morning of every Tuesday and Friday. In the afternoon of the same day, 16 mg pingyangmycin (national drug approval no. H20123357, Jilin Adodong Pharmaceutical Group Yanji Co., Ltd., 8 mg) and 5 mg dexamethasone (national drug approval no. H20044139, Tianjin Jinjin Pharmaceutical Co., Ltd.) were intravenously injected. One treatment cycle was 8 weeks. The basic nursing care included daily saline solution rinsing of patients' mouths, health guidance and nutritional guidance, observation of patients' condition changes, and providing patients with a neat and clean ward environment. In the observation group, patients were treated with TCM anticancer decoction and comprehensive nursing intervention on the basis of the control group. In this study, we reviewed a large amount of literature related to Chinese medicine [22–27], and through the joint research and selection of authoritative Chinese medicine practitioners in our hospital, the prescription of TCM anticancer decoction was 15 g of raw milkvetch root, 15 g of dried fresh ginseng, 15 g of largehead atractylodes rhizome, 15 g of Indian bread, 15 g of pinellia tuber, 15 g of Coix seeds, 15 g of Hedyotis, 15 g of barbated skullcup herb, 10 g of giant knotweed rhizome, 10 g of officinal magnolia bark, 10 g of zedoary rhizome, 10 g of cablin patchouli herb, 6 g of Chinese date, 6 g of fresh ginger, 6 g of golden thread, and 6 g of liquorice root. Add or subtract medicine according to the patient's actual condition. One dose per day, decocted to 200 ml with water, is to be taken warm in the morning and evening.

Oral care: the patient's oral care was rinsed with 0.5% povidone-iodine solution [28], and the patient's head was raised 20° with the head to the side during rinsing, and the patient's teeth, cheek, tongue, pharynx, and hard palate were rinsed slowly in turn, and the fluid in the oral cavity was aspirated with a suction tube during the rinsing process, and the negative pressure of the suction tube was controlled at 0.04 to 0.06 mPa. A long cotton swab could be used to gently wipe the blood crust and oral secretion attachment site until the aspirate became clear. Psychological care: the nursing staff should establish good communication with the patients, actively answer their questions, encourage their family members and friends to give them comprehensive care, support them in life and emotion, enhance their self-worth, and improve their will and self-confidence in facing oral cancer.

### 2.3. Observational Index

Oral cleanliness evaluation standard [29]: grade III referred to ideal oral cleanliness, with a small number of oral bacterial colonies and no foreign bodies on the gums and teeth and no odor; grade II was ideal oral cleanliness, with less oral bacterial colonies, foreign bodies on gums and teeth, and no odor. Grade I referred to poor oral cleanliness, with a large number of oral bacterial colonies, foreign bodies on gums and teeth, and odor. The evaluation standard of sputum aspiration frequency [30]: according to the records of medical staff on the number of sputum aspiration times of patients every day, the minimum sputum was less than 2 min per sputum aspiration time and less than 10 times per day. The moderate sputum volume was 2-5 min for each sputum aspiration and 10-20 times of sputum aspiration per day. Excessive sputum was more than 5 minutes per time of sputum aspiration and more than 20 times per day. Oral comfort evaluation criteria were as follows: nursing staff to understand the feelings of patients by asking patients, according to the real oral cleanliness and comfort of patients into good, good, general three grades. The nursing staff understood the patient's feeling by asking the patient, and according to the actual oral cleanliness and comfort of patients, the oral comfort degree was divided into three grades: good, better, and average.

The immune function of the two groups was compared: 3 ml of peripheral blood of the two groups was collected 2 days before nursing and 8 weeks after nursing, respectively. The levels of T lymphocyte subsets (CD4+, CD8+, and CD56+ cells) in serum of the patients were detected by flow cytometry, and the ratio of CD4+/CD8+ was calculated.

*To compare the levels of tumor markers in the two groups*: enzyme-linked immunosorbent assay (ELISA) was used to measure the expression levels of carcinoembryonic antigen (CEA), neuron specific enolase (NSE), and carbohydrate antigen 19-9 (CA19-9) in the serum of patients before and after care.

*To compare the two-year cumulative survival rates and recurrence rate of the two groups*: after care, all patients were followed up regularly for 2 years, with outpatient or telephone follow-up every 3 months, and the two-year cumulative survival rates and recurrence of the two groups were counted and compared.

*To compare the degree of pain, recovery, and quality of survival between the two groups*: the VAS [31], QoR40 [32], and UW-QOL scale [33] scores were used to assess the degree of pain, quality of recovery, and quality of survival in the two groups, with total scores of 0-10, 40-200, and 0-100, respectively. The quality of recovery and quality of survival gradually improved as the score increased.

The incidence of adverse reactions during care was compared between the two groups, and CTCAE evaluation criteria [34] were used to evaluate reactions including halitosis, oral fungal infections, fever, leukopenia, and gastrointestinal reactions.

*To compare the nursing satisfaction of the two groups*: a homemade questionnaire was filled out at the time of patient discharge with a total score of 100 points and three levels (very satisfied: >90 points, satisfied: >70 points, and unsatisfied: ≤70 points), with higher scores representing better patient satisfaction.

### 2.4. Statistical Analysis

The SPSS 20.0 software was used for statistical analysis of the data. Measurement data were expressed as mean ± standard deviation ($\bar{x}\pm s$), independent sample *t* test was used for inter-group comparison, and paired *t* test was used for intragroup comparison. The count data were expressed as the number of cases and rate (%), and *χ*^2^ test was used for comparison between groups. GraphPad5 was used to draw the survival curve, and log-rank test was used for comparison between groups. *P* < 0.05 was considered as statistically significant difference.

## 3. Results

### 3.1. Comparison of Clinical Efficacy between the Two Groups

The differences between the two groups in oral care effects including oral cleanliness, suction frequency, and oral comfort were statistically significant (*P* < 0.05, Table 2).

### 3.2. Comparison of Immune Function between Two Groups before and after Care

**Figure 1:**
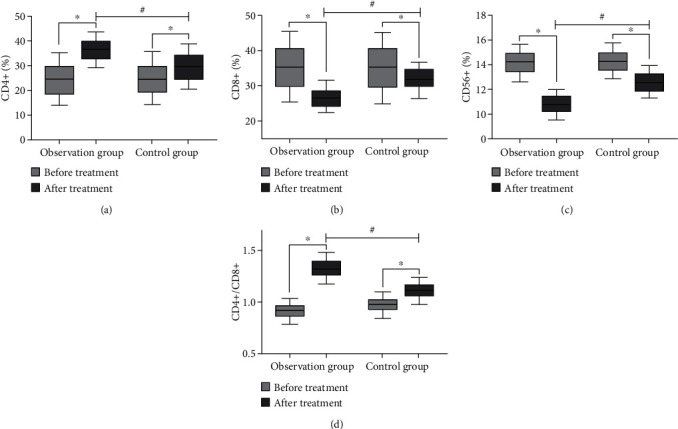
Comparison of immune function between the two groups before and after nursing (^∗^compared with before nursing, *P* < 0.05; ^#^compared with the control group, *P* < 0.05). (a) The comparison of CD4+ level between the two groups before and after nursing. (b) The comparison of CD8+ levels between the two groups before and after nursing. (c) The comparison of CD56+ level between the two groups before and after nursing. (d) The comparison of CD4+/CD8+ between the two groups before and after nursing.

Before care, the differences in CD4+, CD8+, CD56+, and CD4+/CD8+ levels between the two groups were not statistically significant (*P* > 0.05); after care, CD4+ and CD4+/CD8+ levels increased and CD8+ and CD56+ levels decreased in both groups, and the differences were statistically significant (*P* < 0.05, Figure 1).

### 3.3. Comparison of Adverse Reactions between the Two Groups after Nursing

There were statistically significant differences in halitosis, oral fungal infection, leukopenia, gastrointestinal reaction, and fever between the two groups (*P* < 0.05, Table 3).

### 3.4. Comparison of Nursing Satisfaction between the Two Groups

The nursing satisfaction of the observation group was 95.24%, and that of the control group was 78.57%. The difference between the two groups was statistically significant (*χ*^2^ = 8.333, *P* = 0.016) (Table 4).

### 3.5. The Levels of Tumor Markers Were Compared between the Two Groups before and after Nursing

**Figure 2:**
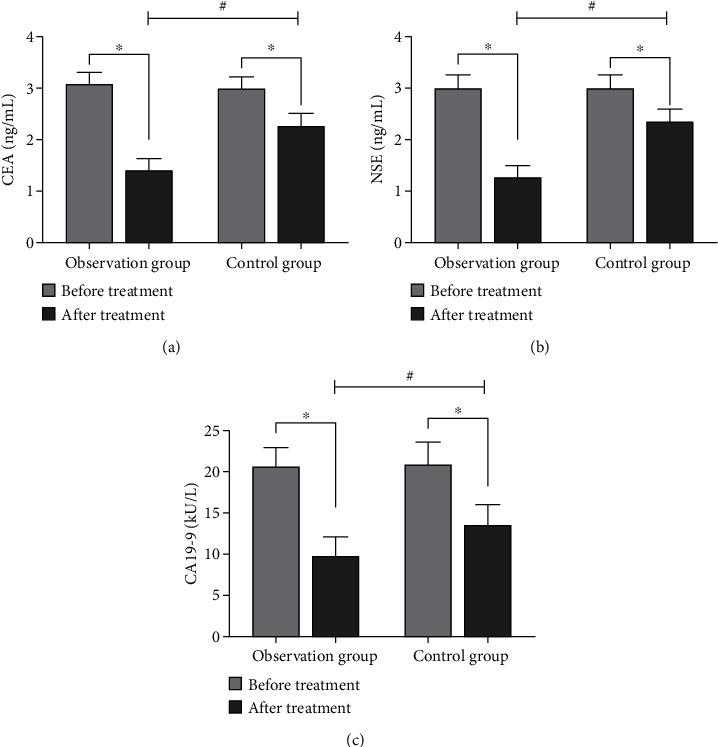
Comparison of tumor marker levels between the two groups before and after nursing (^∗^compared with before nursing, *P* < 0.05; ^#^compared with the control group, *P* < 0.05). (a) The comparison of CEA levels between the two groups before and after nursing. (b) The comparison of NSE levels between the two groups before and after nursing. (c) The comparison of CA19-9 levels between the two groups before and after nursing.

Before nursing, there were no significant differences in CEA, NSE, and CA19-9 levels between 2 groups (*P* > 0.05). After nursing, the levels of tumor markers in both groups were decreased, and the decrease in the observation group was more obvious than that in the control group (*P* < 0.05, Figure 2).

### 3.6. VAS Score, QoR40 Score, and Quality of Life Score Were Compared between the Two Groups before and after Nursing

Before nursing, there were no significant differences in VAS score, QoR40 score, and quality of life between the two groups (*P* > 0.05). After nursing, VAS score, QoR40 score and quality of life score increased in both groups, and the scores of the observation group increased more significantly (*P* < 0.05, Tables 5 and 6).

### 3.7. Comparison of Two-Year Cumulative Survival Rates and Recurrence Rate between the Two Groups

**Figure 3:**
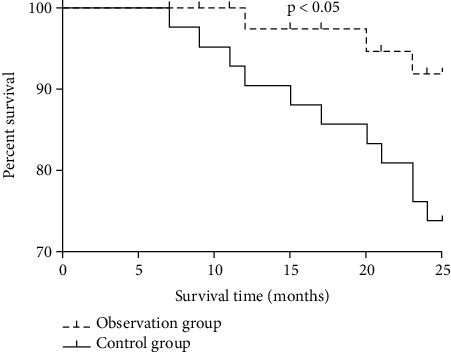
Comparison of two-year cumulative survival rates between the two groups.

The two-year cumulative survival rate was 92.86% in the observation group and 73.81% in the control group (*P* < 0.05, Figure 3). The recurrence rate was 31.0% (13/42) in the control group and 11.9% (5/42) in the observation group. The difference between the two groups was statistically significant.

## 4. Discussion

Oral cancer is a general term for a group of malignant tumors that occur mainly in the oral cavity, mostly squamous epithelial cell carcinoma. At the present stage, patients with oral cancer in China mainly have a high prevalence in the middle-aged and elderly population, but in the late 1970s and especially since the 1980s, there has been a significant ageing trend in oral cancer patients worldwide [35]. According to relevant data [36], patients aged 60 years or older account for about 30% of the total number of patients. In recent years, with the rapid development of oral cancer treatments, the quality of survival of oral cancer patients has been greatly improved and the mortality rate has decreased, but the absolute number of patients is still large because of the large population in China. The goal of tumor treatment is not only to eliminate lesions and reduce recurrence but also to maximize the quality of life of patients and preserve the function of the organism as the goal to be pursued, following the principle that survival rate and quality of survival are equally important [37]. Currently, the use of a single means to treat oral cancer is prone to postoperative recurrence and metastasis, and the long-term efficacy is not ideal, and conducting a comprehensive sequence of various protocols to improve clinical efficacy has been a hot spot of research in recent years. In this study, we used TCM anticancer decoction with nursing intervention to treat postoperative patients with oral cancer.

In this study, raw milkvetch root, dried fresh ginseng, and largehead atractylodes rhizome could strengthen the body and nourish the spleen and kidney. Indian bread could regulate blood and nourish qi, support healthy energy to eliminate evils; zedoary rhizome had the effect of promoting blood circulation and dispersing knot. Hedyotis had the effect of replenishing vital essence to strengthen the kidneys; giant knotweed rhizome could detoxify and fight cancer, soothe liver, and promote blood circulation; Coix seed, pinellia tuber, barbated skullcup herb, cablin patchouli herb, officinal magnolia bark, and golden thread had the functions of expelling phlegm and resolving turbidity, clearing away heat and detoxifying; Chinese date, fresh ginger can spleen stomach, blood tonic; liquorice root played a role in harmonizing various herbs [38–42]. The whole formula played the effect of invigorating the spleen and benefiting the kidney, detoxifying and removing blood stasis.

In this study, after nursing care, the expression levels of tumor markers in the serum of patients in both groups were lower than those before treatment in this group (*P* < 0.05), and the expression levels of tumor markers in the serum of patients in the observation group were significantly lower than those of patients in the control group (*P* < 0.05). CEA, NSE, and CA199 are commonly used serum tumor markers in clinical practice. Studies have shown that the blood levels of tumor markers are significantly higher in patients with malignant tumors than in healthy individuals [43].CEA is a glycoprotein located on the cell surface and is synthesized in early fetal life by the gastrointestinal tract, liver, and pancreas, and in adults, the epithelial tissue of the gastrointestinal tract and the liver and pancreas can also synthesize small amounts of CEA and secrete it into the digestive tract at low levels under normal conditions [44]. There have been many studies on CEA as a marker of oral and maxillofacial malignancies [45, 46]. CEA has been found to be highly expressed in tumor tissues of patients with squamous cell carcinoma of the head and neck [47]. NSE is a glycolytic enzyme present in neuroendocrine cells, neurons, and neurogenic tumors, and its expression is increased in squamous carcinoma tissues with high sensitivity and specificity [48]. CA19-9 is an oligosaccharide tumor-associated antigen first found in gallbladder and pancreatic cancer tissues. Nowadays, its expression has been found to be increased in lung cancer, breast cancer, and other tumor tissues [49–51].

Results of current study indicated that postoperative treatment combined with basic chemotherapy and anticancer decoction together with nursing intervention could effectively eliminate malignant lesion tissues of oral cancer patients. Zedoary rhizome, barbated skullcup herb, and Hedyotis in TCM anticancer decoction had obvious antitumor effects, which could more effectively reduce the expression levels of tumor markers in serum of oral cancer patients after surgery.

The results of this study showed that the oral cleanliness, suction frequency, and comfort of patients in the observation group were significantly higher than those in the control group after care, and the occurrence of adverse reactions was significantly lower than those in the control group. It showed that the combination of TCM anticancer decoction, basic chemotherapy, and comprehensive nursing intervention could improve the therapeutic effect of oral cancer patients. 0.5% povidone-iodine solution could oxidize bacterial active genes and denature proteins after the slow release of active iodine components in oral mucosal tissue rinsing, which could finally achieve the purpose of sterilization. Moreover, it was an effective oral care solution because of its small stimulating effect, long-lasting and strong bactericidal ability, and clear flavor to eliminate oral odor [52]. The abnormal immune function of oral cancer patients, coupled with surgical stress, further reduced the immune capacity. Studies have reported that cellular immunity among the antitumor immune effects of the body can significantly remove tumor cells from the body, among which T lymphocyte subsets and NK cells play a major role, and their concentration changes can objectively reflect the degree of tumor cell suppression [53]. Another study has also been claimed that the kidney is an important organ of reproductive immunity of the body and plays a stabilizing and regulating role in immune function; the spleen also has a significant enhancing effect on immune function [54, 55]. The results of this study showed that the immune capacity of the observation group was significantly higher than that of the control group after the care. This indicated that the combination of TCM anticancer decoction, basic chemotherapy, and nursing intervention could effectively mobilize the immune function of the body and had the effects of strengthening the spleen and stomach, nourishing the liver, and tonifying the kidney. The results of this study showed that the nursing satisfaction, quality of survival, and two-year cumulative survival rate of the observation group were significantly higher than those of the control group, and the recurrence rate was significantly lower than the control group. The control group was only given basic oral rinsing, nutritional guidance, and health guidance, while the observation group was given professional oral rinsing and psychological care on the basis of the control group, since patients would have psychological fear and irritability after diagnosis, and the torture of the disease would make them more overwhelmed. Thanks to the professional guidance and care of nursing staff, the oral condition of patients would be improved, making them feel comfortable and further improving their treatment compliance and nursing satisfaction and the clinical treatment effect. It indicated that the combination of nursing intervention, basic chemotherapy, and TCM anticancer decoction could improve nursing satisfaction and patients' survival quality, effectively kill tumor cells, and significantly improve patients' survival time with higher safety.

In conclusion, TCM anticancer decoction combined with basic chemotherapy and nursing intervention could effectively improve the immune function of postoperative oral cancer patients, reduce tumor cell activity and the occurrence of adverse reactions, improve their clinical efficacy, enhance the survival quality, and prolong the survival time of patients. However, the sample size of this study was small, and the observation period was short, so further clinical research is needed to verify.

## Figures and Tables

**Table 1:** Comparison of general data between the two groups.

	Observation group (*n* = 42)	Control group (*n* = 42)	*χ* ^2^	*P* value
Gender			0.449	>0.05
Male	27	24		
Female	15	18		
Age (years)			0.194	>0.05
>60	23	25		
≤60	19	17		
Degree of education			0.192	>0.05
High school and below	22	24		
College and above	20	18		
Betel nut			0.094	>0.05
Yes	28	30		
No	14	13		
Cigarettes			0.283	>0.05
Yes	34	32		
No	8	10		
Alcohol			0.664	>0.05
Yes	35	32		
No	7	10		
Disease type			0.501	>0.05
Tongue cancer	21	20		
Gingival carcinoma	13	15		
Palate cancer	6	6		
Cheilocarcinoma	2	1		
ASA			0.297	>0.05
I	11	13		
II	26	25		
III	5	4		

**Table 2:** Comparison of oral nursing effect between the two groups.

Grade	Observation group (*n* = 42)	Control group (*n* = 42)	*χ* ^2^	*P* value
Cleanliness of oral cavity			7.863	<0.05
I	2	11		
II	16	15		
III	24	16		
Frequency of sputum suction			6.992	<0.05
More	7	17		
Medium	16	15		
Less	19	10		
Oral comfort			6.424	<0.05
Good	22	11		
Better	14	19		
Average	6	12		

**Table 3:** Comparison of adverse reactions between the two groups.

Group	*n*	Halitosis	Oral fungal infections	Leukopenia	Gastrointestinal reactions	Fever
Observation group	42	3 (7.14)	3 (7.14)	2 (4.76)	4 (8.52)	3 (7.14)
Control group	42	11 (26.19)	9 (21.43)	8 (19.05)	11 (26.19)	6 (14.29)
*χ* ^2^		6.903	6.134	5.773	7.624	5.714
*P* value		<0.05	<0.05	<0.05	<0.05	<0.05

**Table 4:** Comparison of nursing satisfaction between the two groups.

Group	*n*	Very satisfied	Satisfied	Unsatisfied	Degree of satisfaction
Observation group	42	23	17	2	42 (95.24)
Control group	42	12	21	9	33 (78.57)

**Table 5:** Comparison of postoperative VAS and QoR40 scores between the two groups of patients ($\bar{x}\pm s$).

Group	VAS score		QoR40 score	
	Before nursing	After nursing	Before nursing	After nursing
Observation group (*n* = 42)	4.64 ± 1.37	8.41 ± 0.76	91.27 ± 23.55	173.14 ± 18.52
Control group (*n* = 42)	4.58 ± 1.42	6.34 ± 1.03	93.46 ± 22.63	132.68 ± 20.04
*t*	1.623	5.798	2.152	11.663
*P*	>0.05	<0.05	>0.05	<0.05

**Table 6:** Comparison of quality of life between the two groups ($\bar{x}\pm s$).

Group	Material state	Social function	Mental function	Physical function
Observation group (*n* = 42)	57.83 ± 6.71	61.58 ± 5.42	59.26 ± 7.92	64.14 ± 5.73
	84.22 ± 6.29	88.65 ± 6.77	87.97 ± 6.49	86.36 ± 5.67
Control group (*n* = 42)	58.26 ± 6.57	62.14 ± 5.83	59.64 ± 7.58	64.82 ± 5.42
	71.45 ± 6.32	73.48 ± 6.53	72.87 ± 6.62	72.86 ± 5.27
*t*	7.633	8.842	7.964	9.721
*P*	<0.05	<0.05	<0.05	<0.05

## Data Availability

The data used to support the findings of this study are available from the corresponding author upon request.
